# Role of tRNA-derived small RNAs(tsRNAs) in the diagnosis and treatment of malignant tumours

**DOI:** 10.1186/s12964-023-01199-w

**Published:** 2023-07-21

**Authors:** Mingwen Mao, Weina Chen, Xingbiao Huang, Dong Ye

**Affiliations:** 1 Department of Otorhinolaryngology-Head and Neck Surgery, Ningbo No.6 Hospital Affiliated Medical School of Ningbo University, Ningbo, 315040 Zhejiang China; 2 Department of Otorhinolaryngology-Head and Neck Surgery, Lihuili Hospital of Ningbo University, Ningbo, 315040 Zhejiang China; 3 Department of Clinical Pharmacology, Yinzhou Integrated TCM & Western Medicine Hospital, Ningbo, 315040 Zhejiang China; 4 Department of General Surgery, Ningbo No.6, Hospital Affiliated Medical School of Ningbo University, Ningbo, 315040 Zhejiang China

**Keywords:** tsRNAs, Malignant tumour, tRNA-derived fragments, Diagnosis, Treatment

## Abstract

Malignant tumours area leading cause of death globally, accounting for approximately 13% of all deaths. A detailed understanding of the mechanism(s) of the occurrence and development of malignant tumours and identification of relevant therapeutic targets are therefore key to tumour treatment. tsRNAs(tRNA-derived small RNAs)—also known as TRFs (tRNA-derived fragments), tiRNAs (tRNA-derived stress-induced RNAs), tRNA halves, etc.—are a recently identified class of small noncoding RNAs that are generated from mature tRNA or tRNA precursors through cleavage by enzymes such as angiogenin, Dicer, RNase Z, and RNase P. Several studies have confirmed that dysregulation of tsRNAs is closely related to the tumorigenesis of breast cancer, nasopharyngeal cancer, lung cancer, and so on. Furthermore, research indicates that tsRNAs can be used as clinical diagnostic markers and therapeutic targets for cancer. In our review, we summarized the recent research progress on the role and clinical application of tsRNAs in tumorigenesis and progression.

Video Abstract

**Supplementary Information:**

The online version contains supplementary material available at 10.1186/s12964-023-01199-w.

## Introduction

Malignant tumours are currently one of the leading causes of death worldwide, accounting for approximately 13% of all deaths, and their morbidity and mortality rates are increasing [1]. The lack of effective early diagnostic markers and detection methods for malignant tumours means that many patients are already at an advanced stage of disease when they are diagnosed, and the 5-year survival rate is unsatisfactory [2]. It was previously believed that malignant tumorigenesis was due to proto-oncogene activation, resulting in abnormal hyperproliferation of tumour cells;however, recent studies have found that tsRNAs(tRNA-derived small RNAs)—a recently identified class of noncoding small RNAs—are dysregulated in a variety of malignant tumours, thereby affecting the occurrence and development of tumours [3, 4].

tsRNAs—also known as TRFs (tRNA-derived fragments), tiRNAs (tRNA-derived stress-induced RNAs), tRNA halves, etc.—can be expressed in various tissues and organs and are generated from mature tRNAs or tRNA precursors viacleavage by enzymes such as angiogenin, Dicer, RNase Z, and RNase P [5–8]. Several studies have confirmed that tsRNA dysregulation is closely related to the tumorigenesis of breast cancer, nasopharyngeal cancer, lung cancer, and so on. Increasing research on tsRNAs has revealed that these noncoding RNAs can be used as clinical diagnostic markers and therapeutic targets for cancer [9]. This article summarizes the research on the diagnosis, treatment, and mechanism(s) of tsRNAs in malignant tumours.

### Classification, generation, and structure of tsRNAs

#### Classification of tsRNAs

TransferRNA-derived small RNAs (tsRNAs) are small RNA fragments generated from precursor or mature tRNAs, which are precisely cleaved into tRNA loops by specific nucleases (such as Dicer and angiogenin) in specific cells and tissues or under specific conditions [10, 11]. The resulting tsRNAs can be divided into two main types depending on the cleavage site: tRNA-derived fragments (TRFs) and tRNA-derived stress-induced RNAs (tiRNAs) [5, 6].

TRFs are mostly 14–32 nucleotides in length and can be further divided into five subclasses depending on the site of action of the enzyme; these subclasses are TRF-1 s, TRF-2 s, TRF-3 s, TRF-5 s, and i-tRFs [7]. There are three major TRFs: TRF-5, TRF-3, and TRF-1. TRF-5 and TRF-3 are derived from the 5ʹ and 3ʹ ends, respectively, of mature tRNA and are also called 5ʹTRF and 3ʹTRF. TRF-5 is generated by Dicer enzyme cleavage at D-loop or the stem region between D-loop and the anticodon loop, and depending on the cleavage site, TRF-5 can be divided into three isoforms: TRF-5a (the shortest), TRF-5b, and TRF-5c (the longest) [7, 8].

### Generation and structure of tsRNAs

**Fig. 1:**
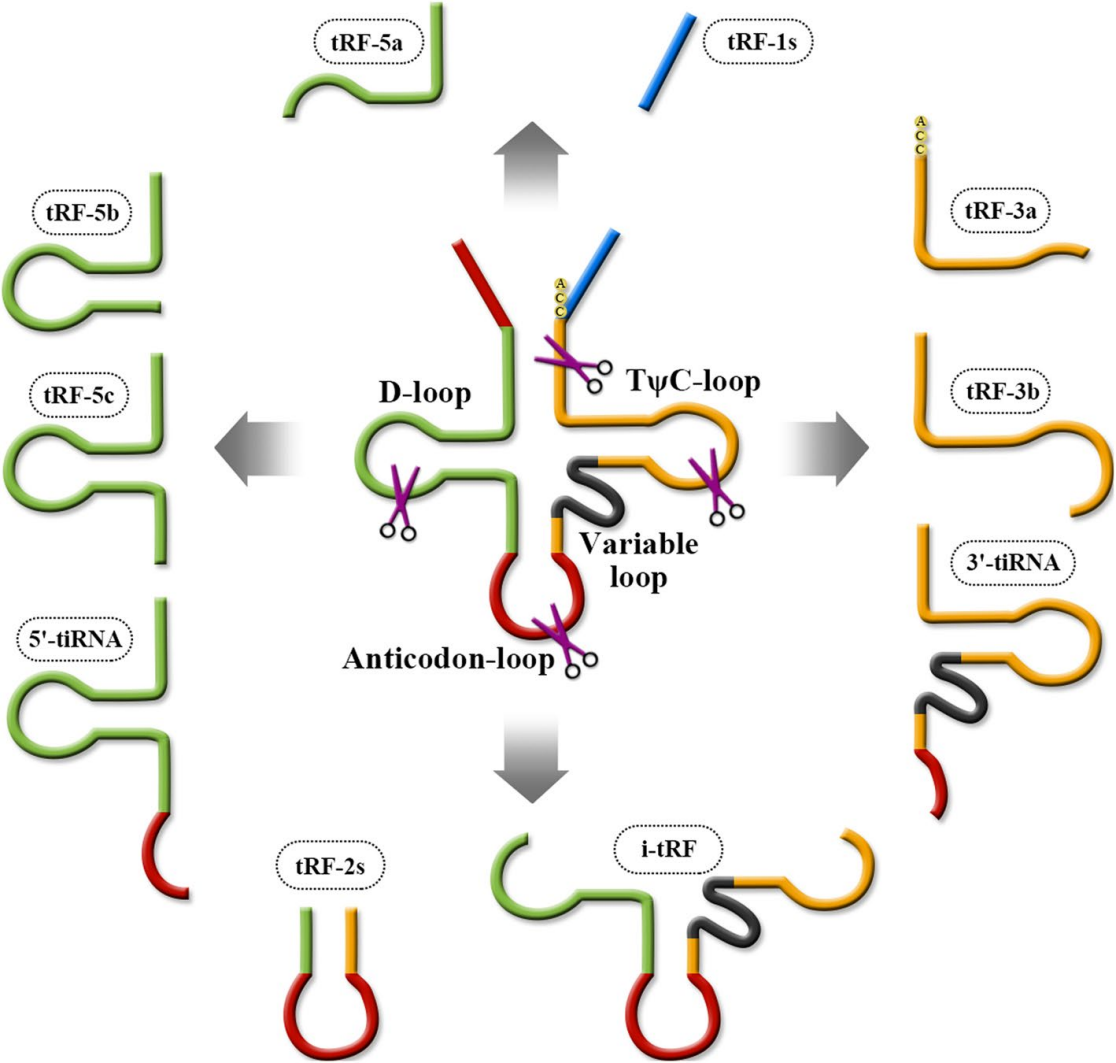
Generation and structure of tsRNAs

tsRNAs are structurally distinct, small RNA fragments generated by precise cleavage of tRNA loops by nucleases such as Dicer and angiogenin. Figure 1 depicts the generation and structure of tsRNAs.

Members of the TRF-1 s subclass are derived from pre-tRNA and contain an RNA polymerase III transcription termination sequence at the 3ʹ end. The first TRF-1 to be discovered was named TRF-1001, and its sequence starts at the 3ʹ end of the mature tRNA, just before the addition of the CCA sequence. There are five or six consecutive thymine bases at the 3ʹ end of the corresponding site, which is a typical termination signal for RNA polymerase III transcription.These sequence features indicate that TRF-1 s are derived from the 3ʹ tail sequence of the pre-tRNA; according to in tRFdb (a database for TRF fragments), each TRF-1 was successively named TRF-1001, TRF-1003, TRF-1004, and so on, in order of discovery [12].

TRF-3 s are derived from the 3ʹ end of the mature tRNA by angiogenin, Dicer, or exonuclease cleavage of the TψC-loop [13], and based on the cleavage position(U/A or U/U), TRF-3 s can be divided into two types: TRF-3a and TRF-3b. The 22-nucleotide TRF-3 results from cleavage between nucleotides 54 and 55 on the TψC-loop, while the 18-nucleotide TRF-3 results from cleavage between nucleotides 58 and 59 on the TψC-loop. The end of this sequence contains the CCA sequence added during mature tRNA processing. The number of TRF-3 s is significantly higher than that of TRF-1 s; 461 TRF-3 s have been identified and recorded in tRFdb [12, 14].

TRF-5 s can be generated by Dicer and angiogenin cleavage, starting at the 5ʹ end of mature tRNA and including the 5ʹ end intact structure, terminating before the anticodon loop [15]. Based on their different termination sites, TRF-5 s can be divided into three isoforms: TRF-5a, TRF-5b, and TRF-5c, which are 14–16, 22–24, and 28–30 nucleotides in length, respectively. The number of TRF-5 s is also significantly higher than that of TRF-1 s and TRF-3 s, with 539 species recorded to date [8].

TRF-2 s/i-tRFs are a class of atypical tsRNAs derived from tRNAs. Novel unclassified tRFs that were first identified in breast cancer cell lines are known as tRF-2. These are derived from tRNAAsp, tRNAGlu, tRNATyr, and tRNAGly,and they comprise anticodon stem loop sequences. i-tRFs arise from cleavage within internal sites of mature tRNAs. Additionally, tRNAhalves, usually detected under stress conditions, arise from angiogenin cleavage within the anticodon loop,which leads to the emergence of i-tRFs that may comprise the anticodon [16, 17].

tRNAhalf molecules (tRNA halves, tiRNAs) are another type of small fragment RNA associated with tRNAs. In response to stress induction, angiogenin is stimulated to promote tRNA cleavage, resulting in the production of tiRNAs. Angiogenin-mediated cleavage of different sites on the anticodon loop of mature tRNA generates tiRNAs of approximately 30–40 nucleotides in length [12]. Additionally, depending on the cleavage site, tiRNAs that contain both the 5ʹ sequence and the anticodon loop are called 5ʹtiRNAs, whereas those that contain both the 3ʹ sequence and the anticodon loop are called 3ʹtiRNAs [14]. There is also a special class of tiRNAs that are only induced by sex hormones and are therefore called sex hormone-dependent tiRNAs (SHOT-RNAs) [8].

### Biological functions of tsRNAs

**Fig. 2:**
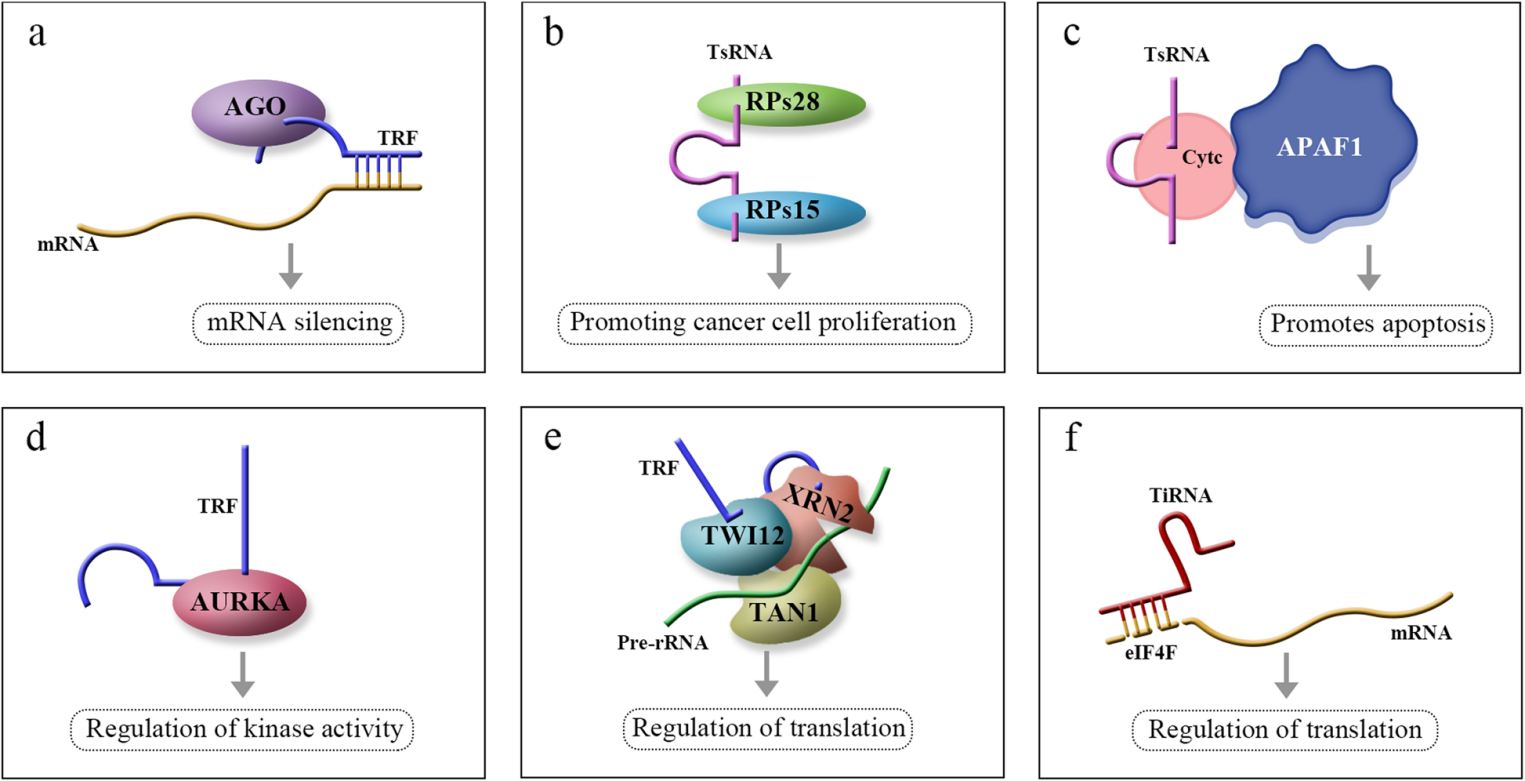
Biological functions of tsRNAs

Although the functions of most TRFs and tiRNAs have not yet been elucidated, increasing evidence suggests that they are involved in the regulation of cell proliferation and apoptosis, gene expression and posttranscriptional modifications, kinase activity, translation, etc. The biological functions of tsRNAs are shown in Fig. 2.

### Regulation of cell proliferation and apoptosis

TRFs may inhibit cell proliferation by regulating target gene expression and posttranscriptional modifications. Certain TRFs function similarly to microRNAs (miRNAs) and piwi-interacting RNAs (piRNAs) and can bind to AGO proteins such as miRNAs or piRNAs to form RISC, which then inhibits mRNA expression [18]. TRF-1 s tend to interact with AGO3 and AGO4 [19, 20], while TRF-5 s and TRF-3 s interact with AGO1, AGO3, and AGO4 [20, 21]. Like miRNAs, TRFs have seed sequences complementary to target mRNAs and can be recruited to AGO complexes to regulate target RNA expression and function [22]. Both TRF-5 and TRF-3 bind to miRNAs through the seed region [23], which has been demonstrated in many other TRFs. For example, ts-53 and ts-101 not only bind to AGO proteins but also to PIWIL2, a piRNA-like molecule, and subsequently regulate target gene expression and posttranscriptional modifications [19, 24]. Research has shown that tRF-3027 (tRNAGly-GCC) may bind to Agronaute (AGO) proteins, which areessential components of RNA-induced silencing complexes (RISCs), and block replication protein A1 (RPA1) to inhibit cell proliferation and regulate the DNA damage response [4, 15, 19]. However, the interaction between tiRNAs and AGO proteins is not clear, and further studies are needed.

tsRNAs are also thought to induce or inhibit apoptosis. A specific tsRNA, 3ʹ-tsRNA-LeuCAG, inhibited the induction of apoptosis in rapidly dividing cells in vitro and in a patient-derived orthotopic hepatocellular carcinoma murine model [18]. Kim et al. found that by binding two or more ribosomal proteins (RPS28 and RPS15), this specific tsRNA promotes cancer cell proliferation. Specifically, inhibition of tsRNALeu − CAG 3'tsRNA induced the apoptosis of hepatocellular carcinoma cells. Inhibition of RPS28 mRNA translation halts the processing of 18S preribosomal RNA, resulting in a decrease in the number of 40Sribosomal subunits [18]. During apoptosis inhibition, tsRNAs and tRNAs have similar roles. Early studies with tRNAs showed that mature tRNAs bind to cytochrome c, inhibit apoptotic body formation and cysteine aspartate protease (caspase-9) activity, and stimulate cell survival [23]. Recent studies have shown that tsRNAs derived from tRNAs also bind to cytochrome c, which then binds to apoptotic protease activating factor 1 (APAF1) to form the apoptosome. In response to hyperosmotic stress, angiogenin mediates the competitive binding of 5ʹ-tiRNA and 3ʹ-tiRNA to cytochrome c, generating a ribonucleoprotein complex that inhibits apoptotic bodies and promotes apoptosis [23, 25].

### Regulation of kinase activity

Shao et al. [26] found that when the expression of tRFLeu − CAG was knocked down, the expression of Aurora kinase A (AURKA) was inhibited. AURKA is a serine threonine kinase that plays an important role in mitosis. It is implicated in centrosome maturation and separationby regulating spindle assembly and stability. Overexpression of tRFLeu − CAG increased the activity of AURKA and then promoted cell cycle progression at the G0/G1 phase in non-small cell lung cancer (NSCLC) [26, 27]. This implies that tRFLeu − CAG promotes tumour cell proliferation by modulating AURKA activity to regulate cell cycle progression.

### Regulation of translation

TRFs can regulate ribosome function. TRF-3 can specifically associate with Twi12, a member of the AGO/PIWI protein family, and promote TAN1 protein and the exoribonuclease XRN2 to form a pre-ribosomal RNA splicing complex (TXT), which processes pre-rRNA during rRNA synthesis and then regulates translation [23]. Recent studies have shown that 5ʹ-tiRNAs (5ʹ-tiRNAala and 5ʹ-tiRNAcys) contain 5ʹ-terminal oligoguanine motifs (5ʹ-TOGs) that may displace eukaryotic translation initiation factor eIF4F at the m7GTP position in mRNA, inhibit translation initiation, and produce multiple mRNA protein complexes (mRNPs) [28]. These tiRNAs may further bind to the cold shock domain (CSD) of YBX-1RNA binding protein to form a 5ʹ-TOG-tiRNA-protein complex, which then stimulates the production of stress granules (SGs) [28]. 5ʹ-tiRNAs may act through phosphorylation of eIF2α to induce assembly of SGs [5, 10, 19, 29]. Another interesting phenomenon is that the functional pattern of TRFs is different from that of typical miRNAs.TRF5-GluCTC is a 5ʹTRF that functions in gene silencing by binding mRNAs complementary to its target sequence. Regulatory studies have shown that the 5′-portion of miRNAs is a key determinant of target recognition and that the 3ʹ-portion of TRF5-GluCTC is essential for its gene silencing function through a trans-silencing mechanism [23]. Unlike miRNAs and piRNAs, TRFs and tiRNAs may directly regulate cellular translation, suggesting that these small RNA molecules have more complex regulatory potential to maintain multiple biological functions and thus play more important roles.

In conclusion, various tsRNAs play a role in translation through diverse mechanisms [5, 10, 23]. For example, they can displace the eukaryotic translation initiation factor eIF4F [28], interact with specific protein factors involved in rRNA synthesis [23], or facilitate the assembly of stress granules (SGs) [19, 29]. These actions exert an impact on crucial stages of the translation process, thereby exerting regulatory control over translation [23, 28].

### tsRNAs in the diagnosis and treatment of malignant tumours

#### Diagnostic role of tsRNAs in malignant tumours

Since tsRNAs were first discovered in the urine and serum of cancer patients in 1979 [30], sequencing technology has revealed tsRNAs in various cancer cells and body fluids. Owing to their stable structure and wide expression, tsRNAs have become potential biomarkers for a variety of tumours [31, 32]. Variations in the expression levels of tsRNAs exist in malignant tumour tissues and exosomes, and these changes in tsRNAs can be used as an indicator for clinical diagnosis and may be associated with the prognosis of tumours. The roles of tsRNAs as clinical diagnostic markers in various large systems are described below.

Shan et al. [33] screened 53 TRFs and tiRNAs from samples of thyroid cancer and normal tissues and found that 19 TRFs and tsRNAs were downregulated and 34 TRFs and tsRNAs were upregulated. qPCR confirmed that trf39-0vl8k87sirmm12e2 exhibitedthe greatest difference in expression between thyroid cancer cells and normal cells. Thus,trf39-0vl8k87sirmm12e2 may serve as a potential biomarker for thyroid cancer. Sequencing of TRFs and tiRNAs identified a 33-nucleotidetiRNAGly that was significantly increased in papillary thyroid cancer(PTC). Ectopic expression of tiRNAGly promoted cell proliferation and migration, while downregulation of tiRNAGly showed the opposite effect. This indicated that tiRNAGly may serve as a PTC biodiagnostic marker [34]. Another study found a total of 158 differentially expressed TRF and tiRNAs in nasopharyngeal carcinoma (NPC), with 88 upregulated and 70 downregulated tsRNAs. Validation of the differentially expressed tsRNAs by qPCR revealed that trf1:28-val-cac-2 had a relatively good ability to discriminate primary NPC from healthy control samples. TRF1:28-val-cac-2 may therefore represent a new class of biological diagnostic markers for NPC [35]. In a study of laryngeal squamous cell carcinoma(LSCC), in which low expression and inhibition of TRF-33q1q89p9l842205 was found in LSCC, receiver operating characteristic (ROC) curve analysis showed that the levels of TRF-33-q1q89p9l842205 could significantly distinguish LSCC tissues from adjacent normal tissues. In addition, the expression level of TRF-33-q1q89p9l842205 is useful for predicting which tumours will develop lymph node metastasis, T stage, and clinical stage. Consequently, TRF-33-q1q89p9l842205 has potential diagnostic value and may be considered a biomarker for LSCC [3, 7].

Pekarsky et al. identified two novel tiRNAs, ts-4521 and ts-3676, which may exhibit antitumour functions in lung cancer. These tiRNAs were downregulated in lung cancer tissues andchronic lymphocytic leukaemia(CLL) [36]. Both tiRNAs have been reported to be involved in apoptosis and chromatin structure,suggesting that they may play a role in regulating tumour cell growth and lung cancer cell survival [37]. It has also been reported that TRF LeuCAG expression is upregulated in human NSCLC, particularly in advanced stages of the disease, and promotes cell cycle progression by targeting AURKA [26]. Collectively, these results indicate that tsRNAs are instrumental in tumour progression and further support that dysregulated tsRNAs of tumours are promising biomarkers for lung cancer diagnosis.

Recent studies have shown that 5ʹTRF GluCTC and 5ʹTRF ValCAC levels are reduced in colorectal cancer cells, suggesting their involvement in colorectal carcinogenesis and their use as potential diagnostic markers [23]. In addition, TRF-Glu-TTC-027 was significantly decreased in gastric cancer (GC) tissues, and TRF-Glu-TTC-027 levels in GC tissues were correlated with features including pathological type, histological grade, and tumour size. Subsequently, the MAPK signalling pathway was confirmed by Western blotting to be the target pathway of TRF-Glu-TTC-027 in GC. This was the first study to show that TRF-Glu-TTC-027 is a novel tumour suppressor and may be a diagnostic biomarker for GC [38]. The expression of tsRNAs in the plasma exosomes of patients with hepatocellular carcinoma (HCC) was significantly higher than that in healthy controls. Four tsRNAs, including tRNA-ValTAC-3, tRNA-GlyTCC-5, tRNA-ValAAC-5, and tRNA-GluCTC-5, were upregulated,suggesting that these differentially expressed tsRNAs have the potential to be novel diagnostic markers for HCC [39].

Analysis of the expression profiles of TRFs revealed that TRF-544 derived from tRNA (Phe)-GAA was downregulated, while TRF-315 derived from tRNA (Lys)-CTT was upregulated in prostate cancer samples vs. control samples. A highratio of TRF-315/TRF-544 in prostate cancer tissues compared with healthy tissues was correlated with poor progression-free survival and shorter disease recurrence, suggesting that the ratio of TRF-315 to TRF-544 may be a potential clinical biomarker for tumour progression in prostate cancer [40]. In patients with clear cell renal cell carcinoma (ccRCC), the circulating levels of 5ʹ-tRNA Arg CCT, 5ʹ-tRNA Glu CTC, and 5ʹ-tRNA Lys TTT were lower than those in control subjects, suggesting that 5ʹ-tRNA hybrids have utility as biomarkers for the diagnosis and prognosis of ccRCC [41]. Differential expression profiling and bioinformatics analysis of tRNA-derived small RNAs in muscle-invasivebladder cancer(MIBC) in a Chinese population study revealed that 91 tsRNAs out of 406 tsRNAs were significantly differentially expressed in MIBC tissues. Thus, MIBC-associated tsRNAs may serve as biomarkers [42].

Upregulation of tsRNA-26576 in tissues from patients with breast cancer was associated with inhibition of cancer cell apoptosis and promotion of cancer cell growth, migration, and invasion. Moreover, the expression of two tumour suppressor genes, *spen* and *fat4*, was increased after downregulation of tsRNA-26576 treatment. Hence,tsRNA-26576 may serve as a therapeutic target for breast cancer [43].

TRF-03357 and TRF-03358 were significantly elevated in serum and cells from patients with high-grade ovarian cancer. In particular, TRF-03357 promotes cancer cell proliferation, migration, and invasion by repressing the tumour suppressor HMBOX1 [44]. Therefore, trf03357 may serve as a potential diagnostic biomarker for high-grade ovarian cancer.

In a study of multiple myeloma, qPCR was employed to detect and analyse the differential expression of TRFs/tiRNAs between healthy donors and multiple myeloma patients. The expression of tiRNA1:34-Glu-ttc-2 was upregulated and that of TRF-60:76-Arg-acg1-m2 was downregulated in multiple bone marrow samples; thus, TRFs/tiRNAs may play a crucial role in the pathogenesis of myeloma and may serve as clinical biomarkers in the future [45].

In summary, some tsRNAs are highly expressed in tumor tissues and serum/plasma of cancer patients [33, 34, 39]. They moderate cell growth by directly or indirectly targeting oncogenic proteins or pathways, and have been strongly associated with tumor growth, invasion, and metastasis [43, 44]. However, some tsRNAs showed lower expression in cancer patients and were reported to elicit tumor-suppressive effects [33, 36, 40]. Thus, dysregulated tsRNAs could be used as biomarkers for the diagnosis and prognosis of different cancers [36, 41, 46].

**Fig. 3:**
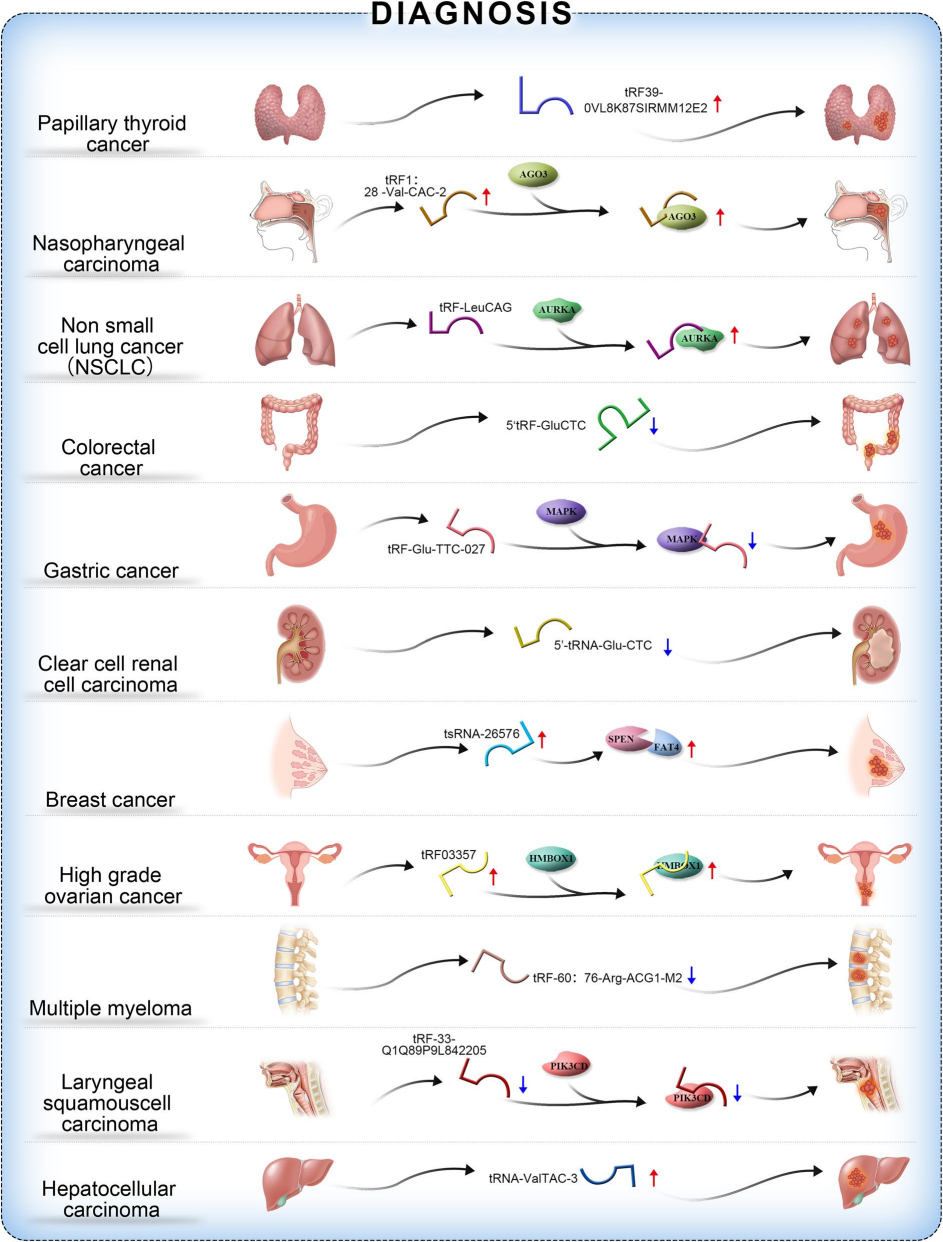
The roles of tsRNAs in the diagnosis of malignant tumours

Figure 3 summarizes current knowledge on the roles of tsRNAs in the diagnosis of malignant tumours.

### Therapeutic role of tsRNAs in malignant tumours

In today’s era of personalized tumour therapy, harnessing immunotherapy to combat cancer is an increasing popular option that can yield significant and durable therapeutic effects across multiple cancers. In recent years, tumour immunotherapy has achieved remarkable success in clinical practice. TsRNAs play both inhibitory and promotionalroles in the initiation and progression of malignant tumours, so they could also theoretically function as clinical therapeutics by altering the expression of corresponding targets. The therapeutic roles of tsRNAs in malignancies are summarized below.

It was recently demonstrated that tiRNAGly acts as a tumour oncogene in PTC. tiRNAGly can bind to the UHM domain of the splicing-related RNA-binding protein RBM17, leading to the translocation and upregulation of rbm17. tiRNAGly exerts its oncogenic effect by inducing rbm17-dependent alternative splicing. These findings provide new insights into the molecular interactions between tRNA fragments and RNA-binding proteins and may contribute to the development of precise approaches for tumour therapy [34]. Denget al. constructed expression profiles of tsRNAs in a study of LSCC and identified a novel 5ʹ-tiRNA fragment (TRF-33-q1q89p9l842205) in LSCC. TRF-33-q1q89p9l842205 was significantly downregulated, and this phenotype was correlated with lymph node metastasis and advanced stages of LSCC. Furthermore, TRF-33-q1q89p9l842205was found toact as a tumour suppressor in LSCC, and PIK3CD was identified as a direct target of TRF-33-q1q89p9l842205 in regulating LSCC progression.Collectively, these findings suggest that TRF-33-q1q89p9l842205 is a potential biomarker for LSCC and possibly acts as a tumour suppressor and therapeutic candidate by directly targeting PIK3CD [46].

Currently, there are very few studies on the role of tsRNAs in lung cancer. Four TRFs (ts-101, ts-53, ts-46, and ts-47) were recently found to be downregulated in lung cancer. Overexpression of ts-46 and ts-47 in two lung cancer cell lines strongly inhibited cell colony formation, confirming that TRFs can affect the growth and survival of lung cancer cells and that ts-46 and ts-47 might be therapeutic targets for lung cancer [37]. The level of TRFLeuCAG in human NSCLC tumour tissues was higher than that in normal tissues, and inhibition of TRF LeuCAG in H1299 cells inhibited cell proliferation, indicating that TRF LeuCAG can serve as a therapeutic target for NSCLC [26]. Studies have found that tsRNA-5001a can accelerate the proliferation of lung adenocarcinoma cells, and high expression of this tsRNA may increase the risk of postoperative recurrence in patients with lung adenocarcinoma. RNA-seq and TCGA database analysis revealed that *GADD45 G*might be the target gene of tsRNA-5001a. Targeting tsRNA-5001a may be a novel approach for the treatment of lung cancer [47].

It was reported that TRF-5026a levels are closely correlated with tumour size and that TRF-5026a inhibits gastric cancer cell proliferation, migration, and cell cycle progression by regulating the PTEN/PI3K/Akt signalling pathway. TRF-5026a may therefore serve as a target for gastric cancer therapy [48]. TRF-1001 is closely associated with the proliferation of colon cancer cells. In the HCT116 cell line, knockdown of TRF-1001 increased the proportion of cells in the G2 phase of the cell cycle and resulted in a significant decrease in cell viability [49]. Subsequently, a comprehensive small RNA sequencing study identified 16 TRFs that were differentially expressed between colon cancer and paired para-carcinoma tissues. Moreover, 55 differentially expressed mRNAs were identified as potential targets of these TRFs [50]. These findings provide valuable clues for further insights into the therapeutic role of TRFs in colon cancer. Enrichment analysis showed that the miRNAs highly correlated with hepatocellular carcinoma tsRNAs were predominantly enriched in fatty acid synthesis and metabolism pathways. Long-chain acyl CoA synthetases (ACSLs), which efficiently activate the most abundant long-chain fatty acids, are known to be commonly deregulated in cancer [51]. Furthermore, fatty acid oxidation provides fuel for metabolic adaptations triggered by β-catenin oncogenic activation in hepatocytes [52]. This suggests that tsRNAs may be regulators that controlfatty acid synthesis and metabolism and could be targets for hepatocellular carcinoma therapy [53].

As previously mentioned,3ʹ-LeuCAGtsRNA induced the apoptosis of rapidly dividing cells in vitro and in a patient-derived orthotopic hepatocellular carcinoma mouse model. This tsRNA binds to at least two ribosomal protein mRNAs (RPS28 and RPS15) to enhance their translation. The reduction in RPS28 mRNA translation prevents pre-18S ribosomal RNA processing, resulting in a reduced number of 40S ribosomal subunits. These data establish that posttranscriptional mechanisms can fine-tune gene expression in different physiological states and provide potential new targets for the treatment of cancer [18].

TRF-1001 is associated with the proliferative capacity of prostate cancer cells. A study showed that silencing the expression of TRF-1001 could arrest prostate cancer cells in the G2 phase, inhibit DNA synthesis, and reduce cell viability and cell proliferation [26, 54]. TRF-1001 thus serves as a therapeutic target for prostate cancer.

In a high-grade serous ovarian cancer (HGSOC) study, sequencing of tRNA-derived small RNA fragment (TRF) and tRNA half (tiRNA) was used to examine the expression profiles of tRNA derivatives in three pairs of HGSOC samples and adjacent normal ovarian tissues. Differentially expressed TRFs and tiRNAs between HGSOC and paired adjacent normal samples were screened. The differentially expressed TRFs and the target genes of the tiRNAs were screened. A total of 20 TRFs and tiRNAs were significantly upregulated and 15 were significantly downregulated in the cancer and para-cancer groups. The biological roles of the upregulated TRFs and tiRNAs included mucin type O-glycan biosynthesis, glycosphingolipid biosynthesis, AMPK signalling pathway, glycosphingolipid biosynthesis, leukocyte transendothelial migration, and starch and sucrose metabolism. The biological roles of the downregulated TRFs and tiRNAs were degradation of other glycans, digestion and absorption of vitamins, fatty acid elongation, and biosynthesis of unsaturated fatty acids. The TRFs and tiRNAs that are significantly upregulated in HGSOC tissues may bepotential diagnostic therapeutic targets for HGSOC [55].

The expression of 5ʹ-tiRNAval was significantly reduced in breast cancer tissues. Serum 5ʹ-tiRNAval downregulation was positively correlated with lymph node metastasis and cancer stage progression, while overexpression of 5ʹ-tiRNAval suppressed the malignant activity of cells. FZD3 was validated as a direct target of 5ʹ-tiRNAval in breast cancer. In addition, in 5ʹ-tiRNAval-overexpressing cells, FZD3, β-catenin, c-myc, and CyclinD1 were downregulated, while APC was upregulated. Furthermore, in breast cancer cells, 5ʹ-tiRNAval inhibited the FZD3-mediated Wnt/β-Catenin signalling pathway. Finally, the sensitivity of the 5ʹ-tiRNAval level in discriminating breast cancer from healthy control samples was 90.0%, and the specificity was 62.7%. This was the first study to identify 5ʹ-tiRNAval as a novel tumour suppressor that acts through inhibition of FZD3/Wnt/β-Catenin signalling, which could be a potential therapeutic target in breast cancer [56]. Recently, tRF-Glu49 expression was found to be significantly downregulated in cervical cancer, and studies have shown that tRF-Glu49 inhibits cervical cancer proliferation, migration, and invasion by targeting fibrinogen-like protein 1(FGL1). These results suggest that tRF-Glu49 may be a promising new target for patients with cervical cancer [57].

Veronicbalatti and colleagues conducted a series of systematic studies on the association of tsRNAs with cancer and demonstrated that tsRNA-53 inhibited the expression of T-cell leukaemia/lymphoma 1 (TCL1) by targeting the untranslated region (UTR) of TCL1 mRNA in B-cell CLL. Therefore, downregulation of tsRNA-53 may lead to increased expression of TCL1 and thus promote CLL progression; consequently, tsRNA-53 may serve as a therapeutic target in CLL [9, 46, 58].

The tRNA-derived fragments tdr-0009 and tdr-7336 were shown to be upregulated in response to doxorubicin in triple-negative breast cancer, and ts-57 s and ts-46 s have also been found to be associated with the resistance of breast cancer to lapatinib. Thus, trd-0009, tdr-7336, ts-57 s, and ts-46 s may be future therapeutic targets in breast cancer [59].

**Fig. 4:**
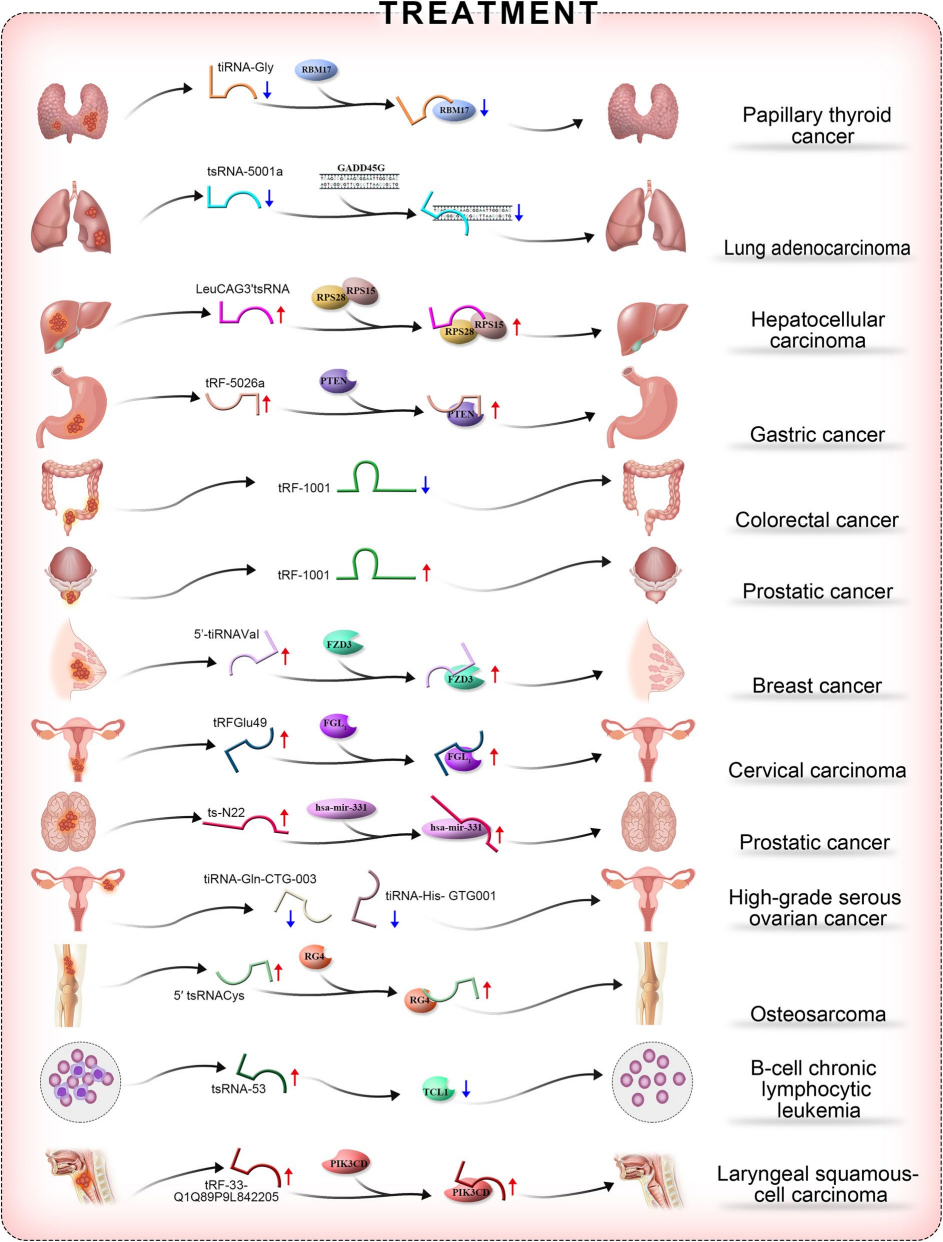
The roles of tsRNAs in the treatment of malignant tumours

Figure 4 summarizes current knowledge on the roles of tsRNAs in the treatment of malignant tumours.

### Possible regulatory mechanisms of tsRNAs in malignant tumours

A growing body of research suggests that tsRNAs play a plasticity regulatory role in cancer cells [43, 44, 54]. Cancer cellular plasticity plays pivotal roles in cancer initiation, progression, therapy resistance and cancer relapse [46, 55, 56, 59]. The regulatory mechanisms of tsRNAs on plasticity of cancer cellular are summarized below.

### tsRNAs regulate cell proliferation and apoptosis in malignant tumours

TRFs and tiRNAs are important regulators of rRNA and protein biogenesis [14, 15, 26]. Kim et al. found that two or more ribosomal proteins (RPS28 and RPS15) − CAG 3ʹtsRNAs promote translation of their target mRNAs, thereby promoting cancer cell proliferation. Specifically, inhibition of LeuCAG3ʹ-tsRNA induced the apoptosis of hepatocellular carcinoma cells. Inhibition of translation of RPS28 mRNA halts the processing of 18S pre-ribosomal RNA, leading to a reduction in the levels of some 40S ribosomal subunits [18].

TRFs and tiRNAs regulate gene expression by binding to RNA-binding proteins (RBPs). Goodarzi et al. showed that TRF-derived tRNAAsp, tRNAGlu, tRNATyr, and tRNAGly and some endogenous oncogene transcripts in breast cancer cells compete for binding with Y-box binding protein 1 (YBX1). YBX1 is an RNA-binding protein with diverse biological functions. YBX1 maintains its stability by binding to some endogenous oncogene transcripts, thereby increasing malignant tumour cell proliferation. Conversely, YBX1 inactivation leads to cell death. When the YBX1 oncogene transcription complex is dissociated, the stability of the oncogene transcription complex is perturbed. At this time, the expression of oncogenes is reduced, and the growth of cancer cells is inhibited. YBX1 can bind multiple types of transcripts, including TRFs and tiRNAs [26].

TRFs and tiRNAs regulate kinase activity. Shao et al. showed that tRFLeu − CAG regulates cell proliferation and cell cycle progression in NSCLC, and when the expression of tRFLeu − CAG was knocked down, the expression of AURKA was also inhibited. AURKA is a serine threonine kinase that plays an important role in mitosis; it is implicated in centrosome maturation and separation, thereby regulating spindle assembly and stability. Overexpression of tRFLeu − CAG increased the activity of AURKA and subsequently promoted cell cycle progression at the G0/G1 phase in NSCLC [23, 26, 27]. This implies that tRFLeu − CAG promotes tumour cell proliferation by modulating AURKA activity to regulate cell cycle progression.

### tsRNAs regulate malignant cell invasion and metastasis

Angiogenin, a member of the ribonuclease A superfamily, not only activates endothelial cells and induces tumour angiogenesis but also targets tumour cells to promote cell migration and invasion [60]. Li et al. showed that angiogenin cleaves tRNAs to generate tiRNAs, which may directly regulate cell migration and invasion by binding to adhesion-related proteins or may affect the expression of key proteins by regulating mRNA splicing. 5ʹ-tiRNA Val was found to be elevated in colorectal cancer (CRC) tissues and serum and correlated with angiogenin levels and CRC invasion and metastasis but had no effect oncell proliferation, thereby establishing a migration and invasion regulatory axis for angiogenin-tiRNAs in CRC cells [61].

ts-n22 is a tsRNA with protective properties in hepatocellular carcinoma(HRZ0.51), and patients expressing ts-n22 had a higher survival rate [53]. The associated factors hsa-mir-331 and hsa-mir-33a are tumour suppressors;hsa-mir-331 could regulate neurocortin-2 expression and inhibit glioblastoma cell metastasis [53].

A recent study of a DT tumour (rectal cancer) demonstrated reduced expression levels of TRF/mir-1280. Furthermore, TRF/mir-1280 inhibits the 3ʹ-UTR of JAG2, reduces JAG2 biosynthesis, inhibits the Notch pathway, and directly inhibits the migration and epithelial–mesenchymal transition (EMT) of rectal cancer cells [23, 62]. TRF/miR-1280 also reduced the expression of CD133 + stem cell markers in CRC cells, reduced their activity and metastatic ability, and prevented the formation of a metastatic microenvironment in rectal cancer cells [62].

### tsRNAs regulate malignant tumour sensitivity to chemotherapeutic drugs

TRFs and tiRNAs may affect the resistance of cancer cells to chemotherapeutic drugs. For example, the tRNA-derived fragments tdr-0009 and tdr-7336 were upregulated and associated with increased chemoresistance to doxorubicin in triple-negative breast cancer [32], while ts-57 s and ts-46 s were associated with the chemoresistance of breast cancer to lapatinib [32]. Furthermore, tRNA-derived fragments may increase chemoresistance by inhibiting eukaryotic translation repressor 4 g [4], which can block the expression of adenosine triphosphate binding cassette (ABC) transporters. These transporters are important for enabling anticancer drugs to cross the cell membrane. In addition, TRFs and tiRNAs generate SGs that can render glioblastoma cells resistant to the anticancer drug bortezomib [3, 63].

Sun et al. comprehensively analysed tRNA-derived fragments in trastuzumab-sensitive and trastuzumab-resistant breast cancer and speculated that tRF-30-JZOYJE22RR33 and tRF-27-ZDXPHO53KSN may participate in trastuzumab resistance by regulating the expression product of target genes or competing with mRNAs for binding to RNA-binding proteins. The mechanism underlying the effects of tRNA-derived fragments on trastuzumab resistance is extremely complicated, and further research is needed [64].

## Conclusion

Although the existence of tRNA breakdown products as cancer markers has been appreciated since the 1970s, the exact function of tsRNAs in cancer has only recently become the subject of intense research. The application of high-throughput sequencing technology has revealed aberrant expression of multiple tsRNAs in multiple cancer types. Some of these tsRNAs have been collated and deposited in several databases. Their aberrant expression indicates their potential as biomarkers. However, most related experiments are based on cell and tissue research. Ideal and effective molecular markers should be stably expressed in serum, plasma, and other body fluids. Such molecules have greater potential for clinical applications.

In the future, the biological function of tsRNAs and the regulatory mechanism in tumorigenesis and development need to be further elucidated.For example, the relationship between the structure of different types of tsRNAs and their functions in tumour biology and the exact relationship between tsRNAs and miRNAs need to be further elucidated. Because they are both noncoding RNAs with theability to combine with AGO proteins, they may have a similar function in regulating gene expression by inhibiting target mRNAs.As research in this area continues, it is believed that the details regarding tsRNAs involved in the regulation of the malignant tumour process will be clarified, which will provide an effective means to further understand the occurrence and progression of tumours and assist in clinical cancer diagnosis and treatment. The roles and possible mechanisms of tsRNAs in malignant tumours are shown in Table 1. In the future, it is expected that tsRNAs will be widely used to facilitate early detection and diagnosis of tumours, help judge prognosis and therapeutic effects, and develop new antitumour drugs and clinical tumour interventions. Finally, studies related to malignant tumour-derived tsRNAs, which reflect the idea and mode of translational medicine research, will greatly advance biomarker research and translational medicine.

**Table 1:** The roles and possible mechanisms of tsRNAs in malignant tumors

Cancer types	tsRNA	type	Expression level	Biological function	Downstream regulators	Target molecules	Predictive use	References
Papillary thyroid cancer	tRF39- 0VL8K87SIRMM12E2	tRF	UP	Regulation of tumor cell proliferation	—	—	Biomarkers for diagnosis	[34]
Papillary thyroid cancer	tiRNA-Gly	tiRNA	UP	Regulation of tumor cell proliferation and migration	RBM17	MAP4K4	Biomarkers for diagnosis and targeted therapy	[35]
Nasopharyngeal carcinoma	tRF1: 28 -Val-CAC-2	tRF	UP	Regulation of tumor cell proliferation and migration	AGO3	—	Biomarkers for diagnosis	[36]
Laryngeal squamous cell carcinoma	tRF-33-Q1Q89P9L842205	tRF	UP	Regulation of tumor cell proliferation and invasion, migration	PIK3CD	—	Biomarkers for diagnosis and targeted therapy	[37]
Lung adenocarcinoma	tsRNA-5001a	tRF	UP	Regulation of tumor cell proliferation	GADD45G	—	Biomarkers for diagnosis and targeted therapy	[48]
Non small cell lung cancer (NSCLC)	tRF-LeuCAG	tRF	UP	Regulation of tumor cell proliferation and cell cycle	AURKA	—	Biomarkers for diagnosis and targeted therapy	[27]
Non small cell lung cancer (NSCLC)	AS-tDR-007333	tRF	UP	Regulation of tumor cell proliferation and migration	HSPB1/ELK4	MED29	Biomarkers for diagnosis and targeted therapy	[65]
Hepatocellular carcinoma	LeuCAG3'tsRNA	tRF	Down	Regulation of tumor cell proliferation	RPS28/RPS15	—	Biomarkers for diagnosis and targeted therapy	[18]
Gastric cancer	tRF-5026a	tRF	Down	Regulation of tumor cell proliferation, migration, and cell cycle progression	PTEN/PI3K/AKT	—	Biomarkers for diagnosis and targeted therapy	[49]
Gastric cancer	tiRNA-Val-CAC-001	tiRNA	Down	Regulation of tumor cell proliferation and migration	LRP6	—	Biomarkers for diagnosis	[66]
Colorectal cancer	5’-tiRNA-Val	tiRNA	Up	Regulation of tumor cell invasion and metastasis	—	—	Biomarkers for diagnosis and targeted therapy	[62]
Gastric cancer	tRF-Glu-TTC-027	tRF	Down	Regulation of tumor cell proliferation and invasion and metastasis	MAPK	—	Biomarkers for diagnosis and targeted therapy	[40]
Prostatic cancer	tRF-1001	tRF	Down	Regulation of tumor cell proliferation	—	—	Biomarkers for diagnosis and targeted therapy	[28, 55]
Breast cancer	5’-tiRNAVal	tiRNA	Down	Regulation of tumor cell proliferation	FZD3	—	Targeted therapy	[57]
Breast cancer	tsRNA-26576	tRF	Up	Regulation of tumor cell proliferation and invasion and metastasis	SPEN/FAT4	—	Biomarkers for diagnosis	[45]
Breast cancer	Ts-57 s、ts-46 s	tRF	Up	Regulation of chemoresistance in tumor cells	—	—	Targeted therapy	[60]
High grade ovarian cancer	tRF03357	tRF	Up	Regulation of tumor cell proliferation, migration and invasion	HMBOX1	—	Biomarkers for diagnosis	[46]
Cervical carcinoma	tRFGlu49	tRF	Down	Regulation of tumor cell proliferation, migration and invasion	FGL1	—	Biomarkers for diagnosis and targeted therapy	[58]
High-grade serous ovarian cancer (HGSO)	tiRNA-Gln-CTG-003、tiRNA-His- GTG001	tiRNA	Up	Regulation of tumor cell proliferation	—	—	Biomarkers for diagnosis and targeted therapy	[56]
Glioblastoma cells	ts-N22	tRF	Down	Regulation of tumor cell metastasis	Hsa-mir-331/ Hsa-mir-33a	Neurocortin-2	Targeted therapy	[54]
Osteosarcoma	5′ tsRNAAla, 5′ tsRNACys	tRF	Down	Regulation of tumor cell proliferation	RG4	—	Targeted therapy	[9]
B cell chronic lymphocytic leukemia (CLL)	tsRNA-53	tRF	Down	Regulation of tumor cell proliferation	TCL1	—	Targeted therapy	[59]
Multiple myeloma	TRF-60:76-Arg-acg1-m2	tRF	Down	Regulation of tumor cell proliferation	—	—	Biomarkers for diagnosis	[47]

## Data Availability

Not applicable.

## Abbreviations

tsRNAs: TRNA-derived small RNAs
TRFs: TRNA-derived fragments
tiRNAs: TRNA-derived stress-induced RNAs
intRFdb: A database for TRF fragments
SHOT-RNA: Sexhormone-dependent tiRNAs
AGO: Agronaute
RISCs: RNA-induced silencing complexes
RPA1: Replication protein A1
caspase-9: Cysteine aspartate protease
APAF1: Apoptotic protease activating factor 1
piRNAs: Piwi-interacting RNAs
AURKA: Aurora kinase A
NSCLC: Non-small cell lung cancer
TXT: Pre-ribosomal RNA splicing complex
mRNPs: MRNA protein complexes
CSD: Cold shock domain
SGs: Stress granules
PTC: Papillary thyroid cancer
NPC: Nasopharyngeal carcinoma
LSCC: Laryngeal squamous cell carcinoma
ROC: Receiver operating characteristic
CLL: Chronic lymphocytic leukemia
GC: Gastric cancer
HCC: Hepatocellular carcinoma
CCRCC: Clear cell renal cell carcinoma
MIBC: Muscle-invasivebladder cancer
ACSLs: Long-chain acyl CoA synthetases
HGSOC: High-grade serous ovarian cancer
FGL1: Fibrinogen-like protein 1
TCL1: T-cell leukemia/lymphoma 1
UTR: Untranslated region
RBPs: RNA-binding proteins
YBX1: Y-box binding protein 1
CRC: Colorectal cancer
DT tumor: Rectal cancer
EMT: Epithelial–mesenchymal transition
ABC: Adenosine triphosphate binding cassette
